# The role of PET/CT in preoperative decision-making for secondary cytoreduction in recurrent ovarian cancer

**DOI:** 10.1186/s12885-026-15898-3

**Published:** 2026-03-26

**Authors:** Csaba Csikos, Antal Varga, Szabolcs Molnár, Zsolt Hascsi, Ferenc Bátyi, Ildikó Garai, Zoárd Tibor Krasznai

**Affiliations:** 1 Division of Nuclear Medicine and Translational Imaging, Department of Medical Imaging, Faculty of Medicine, University of Debrecen, Debrecen, H-4032 Hungary; 2 Gyula Petrányi Doctoral School of Clinical Immunology and Allergology, Faculty of Medicine, University of Debrecen, Debrecen, H-4032 Hungary; 3 Department of Obstetrics and Gynaecology, Faculty of Medicine, University of Debrecen, Debrecen, H-4032 Hungary; 4 Scanomed Ltd., Debrecen, H-4032 Hungary

**Keywords:** Recurrent ovarian cancer, Secondary debulking surgery, Complete cytoreduction, [^18^F]FDG, PET/CT, Preoperative prediction

## Abstract

**Background:**

Secondary cytoreductive surgery is a feasible treatment option in recurrent ovarian cancer. Patient selection for surgery remains challenging, as conventional imaging, such as CT and MRI, is unreliable for predicting complete gross resection. PET/CT is a modality that combines metabolic and anatomical imaging. Although there are studies supporting the use of PET/CT in ovarian cancer, there is very limited evidence specifically assessing PET/CT-guided selection for secondary cytoreductive surgery under the latest eligibility criteria. This study aims to evaluate the added value of PET/CT for predicting no residual tumor volume after secondary surgical cytoreduction.

**Methods:**

We conducted a retrospective data collection on patients diagnosed with ovarian cancer who underwent a PET/CT examination between 2018 and 2023 in our center. Patients with suspected recurrence considered for secondary cytoreduction based on conventional imaging and clinical criteria, were included in our analysis. Besides descriptive statistics we performed decision curve analysis (DCA) to determine the added value of PET/CT in surgical decision-making.

**Results:**

22 patients were potentially eligible for secondary debulking according to clinical criteria and conventional imaging findings. 13/22 patients were found to be operable based on preoperative PET/CT results, and 11/13 had a successful resection (no gross residual tumor) (PPV: 85%). 9/22 patients were found ineligible for secondary cytoreduction based on PET/CT results. 7/9 had inoperable lesions, and in two cases, no malignant lesions were detected. Decision curve analysis showed that PET/CT provided superior clinical utility compared to both conventional imaging and a “treat none” approach, yielding a higher net benefit across a broad range of surgical decision thresholds.

**Conclusions:**

Our results demonstrate that PET/CT adds significant clinical value in selecting appropriate candidates for secondary cytoreduction, mainly by reinforcing potential operability. These findings suggest that PET/CT may provide incremental information during preoperative assessment; however, prospective validation is required.

**Supplementary Information:**

The online version contains supplementary material available at 10.1186/s12885-026-15898-3.

## Introduction

Ovarian cancer is one of the most aggressive types of gynecologic malignancies, with approximately 300,000 new cases and 200,000 deaths reported annually worldwide [1]. Its relatively high mortality rate can be attributed to the fact that most patients are diagnosed at an advanced stage (FIGO III–IV), resulting in a relapse rate of 70–80% [2–5].

Secondary cytoreductive surgery (SCS), also known as secondary debulking surgery (SDS), is a treatment approach that can extend progression-free and overall survival in selected patients when complete cytoreduction is achieved. The role of secondary cytoreductive surgery in platinum-sensitive recurrent ovarian cancer has been investigated in three randomized phase III trials — DESKTOP III, GOG-0213, and SOC-1 — with partially conflicting results [6–9]. The combined results of these trials highlight that the benefit of secondary cytoreduction largely depends on optimal patient selection and the probability of achieving complete resection. Traditional clinical models, such as the AGO score or iMODEL, rely on clinical and surgical variables but do not capture the metabolic characteristics of tumor spread [6–8]. For example iMODEL score is based on FIGO stage at initial diagnosis, residual disease after primary surgery, progression-free interval, performance status (ECOG), CA-125 level at recurrence, and presence of ascites at recurrence [7].

In this context, PET/CT imaging offers complementary anatomic and metabolic information, improving the detection of occult or disseminated disease and helping to exclude patients with unresectable relapse. Building on the SOC-1 experience, PET/CT-based selection may refine surgical candidacy beyond traditional clinical scores and ensure that secondary debulking is offered to patients with truly resectable, biologically favorable disease [7].

According to ESGO-ESMO-ESPC consensus document and the ESGO pocket guidelines based on this document, SDS is a feasible treatment option for adult women diagnosed with ovarian cancer of high-grade serous histology (HGSOC), but the operation is not restricted to high grade serous subtype. In cases of different subtypes, the operation may also be considered [10]. The criteria used in DESKTOP III are generally accepted today for the secondary cytoreductive operation: good performance status (Eastern Cooperative Oncology Group [ECOG] score 0–1), complete cytoreduction at previous operation, and at least a 6-month platinum-sensitive progression-free survival period after primary or interval debulking surgery [5, 6, 10–15]. The recent ESGO-ESMO-ESPC consensus document for ovarian cancer also states that secondary cytoreductive surgery should be considered only for patients with > 6 months' interval after completion of platinum-based chemotherapy and calls for the use of prospectively validated algorithms to identify eligible candidates for the operation [10].

Surgical completeness according to today’s standards is determined by the operating surgeon, and the definition of complete cytoreduction is when no macroscopic tumor remains at the end of the operation [16–18]. It is important to note, however, that the benefit of surgery diminishes substantially when residual disease remains, making patient selection for operability a critical clinical decision. Inappropriate selection may lead to non-beneficial surgeries, with significant morbidity and no oncologic gain [6–9].

Traditionally, conventional imaging modalities, including contrast-enhanced computed tomography (CT) and magnetic resonance imaging (MRI), have been used to determine operability. However, these techniques are often limited in their ability to detect small-volume peritoneal metastases and lymph node involvement, leading to inaccurate assessment of disease extent and operability [19].

[^18^F]fluorodeoxyglucose ([^18^F]FDG) positron emission tomography/computed tomography (PET/CT) is a highly sensitive modality that combines metabolic and anatomical imaging to detect abnormalities. Due to its remarkable sensitivity, it has shown potential to refine patient selection by identifying inoperable conditions that conventional imaging modalities cannot detect [20, 21]. According to the ESGO-ESMO consensus PET/CT may help identify distant sites when planning secondary cytoreductive surgery, but it is not a standard imaging modality of SCS planning [10].

Despite its advantages, [^18^F]FDG PET/CT has important limitations that are particularly relevant when assessing operability in recurrent ovarian cancer. Its sensitivity is reduced for small-volume peritoneal disease, especially implants below the spatial resolution threshold of approximately 7 mm or in the presence of diffuse miliary spread. Furthermore, FDG uptake varies by histologic subtype, with lower metabolic activity frequently observed in low-grade serous, mucinous, and sex cord–stromal tumors, potentially leading to false-negative findings. Conversely, false-positive uptake may occur due to inflammatory processes, postoperative changes, or benign reactive lymph nodes, which may complicate the interpretation of disease extent in the preoperative setting [22].

In spite of these limitations, our recently published systematic review and meta-analysis suggested that PET/CT, when used preoperatively, consistently yields a high positive predictive value for achieving surgical completeness [23].

The majority of previous studies examining the use of PET/CT in patient selection for cytoreductive operations have focused on patients undergoing primary debulking surgery, or did not differentiate between primary and secondary debulking [23]. Most of the limited number of previous publications on PET/CT in secondary debulking do not meet today's standard criteria for a > 6-month platinum-free interval, < 500 ml of ascites, or for the definition of complete tumor resection, which differs from today's “no visible tumor” criterion [24–26].

According to these previous data, a favorable PET/CT result that opts for operability increases the probability of achieving complete secondary cytoreduction. Therefore, PET/CT provides additional valuable information about disease extent, thus aiding patient selection and surgical decision-making.

In this retrospective single-center study, we aimed to assess the added clinical value of PET/CT in the preoperative evaluation of patients with recurrent ovarian cancer who were considered for SCS by primary conventional imaging results, and meet the eligibility criteria of today’s standards for SCS. Our goal was to determine whether PET/CT can improve patient selection by detecting ineligible patients, increasing the rate of complete cytoreduction, and avoiding unnecessary surgeries.

## Methods

### Eligibility criteria

We retrospectively analyzed patients who had histologically proven ovarian cancer and underwent [^18^F]FDG PET/CT examination at our nuclear medicine center between 2018 and 2023. Patient data were obtained from the electronic medical record of the University of Debrecen (UD MED). Patients with suspected recurrence being potentially eligible for secondary debulking based on conventional imaging (i.e. solitary or oligometastatic disease), and meeting all clinical criteria were included in our study.

Clinical inclusion criteria were:

1. Age above 18 years;
2. Potentially eligible for secondary debulking based on conventional imaging;
3. Complete cytoreduction at previous primary/interval debulking surgery (based on operative report);
4. At least six months of progression-free survival following postoperative chemotherapy;
5. ECOG performance state 0 or 1.
6. Multidisciplinary tumor board decision on secondary debulking surgery.

Exclusion criteria were:

1. Incomplete cytoreduction at previous operation;
2. Definitive unresectable tumor on conventional imaging due to the location or multiple complexity of the recurrence based on the decision of a multidisciplinary tumor board.
3. Estimated ascites volume > 500 mL.

All operations were performed in the study center, which is a tertiary care university hospital with a designated gyneco-oncosurgical unit. All operations were performed by gynecologic oncologic surgeons. Complete surgical cytoreduction (R0 resection, no gross residual disease) was declared if the operating gynecologic oncologist could remove all macroscopically visible tumor tissue.

This retrospective study was designed to reflect real-world preoperative decision-making for secondary cytoreductive surgery regardless of histology. Therefore, histology was not used as an inclusion/exclusion criterion, and patients with different ovarian cancer histologic subtypes were included, consistent with the routine clinical practice.

### PET acquisition protocol

Before the examination, capillary blood glucose levels were measured from a fingertip sample. The upper limit for proceeding with the scan was 12.5 mmol/L (225 mg/dL).

[^18^F]fluorodeoxyglucose ([^18^F]FDG) was administered intravenously. The injected activity was weight-based, calculated as 3.5 MBq per kilogram of body weight. A deviation of ± 10% from the calculated activity was considered acceptable, with a maximum administered activity not exceeding 530 MBq.

Immediately prior to radiotracer injection, patients received one ampoule (20 mg) of furosemide intravenously along with oral potassium supplementation. The purpose of diuretic administration was to promote bladder emptying, thereby improving visualization of pelvic structures. In addition, 1 L of diluted oral contrast agent (Gastrografin®; meglumine diatrizoate and sodium diatrizoate) was administered routinely to enhance the precision of localization on low-dose CT images.

Image acquisition was initiated 60 min (± 5 min) after tracer administration. Patients were positioned supine with their arms raised, and scans were acquired from the skull base to the upper third of the thighs. PET data acquisition parameters varied based on the PET/CT system used (Philips Vereos, Philips Gemini TF or Mediso PET/CT) and the patient’s body mass index (BMI). Low-dose CT was performed for attenuation correction and anatomical localization.

Board-certified nuclear medicine physicians who are experienced in reading gynecologic oncologic PET/CTs evaluated and reported the images. PET/CT examinations were interpreted in accordance with current clinical reporting standards. Image analysis was performed by physicians who were aware of relevant clinical information and the results of prior conventional imaging, reflecting routine multidisciplinary clinical practice. Structured reporting was applied in all cases. In instances of equivocal or ambiguous findings, images were reviewed jointly and discussed until a consensus interpretation was reached.

### Statistical analysis

Descriptive statistics were used for demographic data analysis.

Decision curve analysis (DCA) was conducted to compare the net benefit of conventional imaging and PET/CT in preoperative surgical decision-making [27]. The following formula was used to calculate the net benefit across different threshold probabilities:

$$\begin{aligned} \mathrm{Net}\;\mathrm{Benefit}=&\:\left(\mathrm{True}\;\mathrm{Positive}\;\mathrm{Count}\;/\;\mathrm{N}\right)\;\\&-\;\left(\mathrm{False}\;\mathrm{Positive}\;\mathrm{Count}\;/\;\mathrm{N}\right)\;\\& \times\;\left[\mathrm{pt}\;/\;\left(1\;-\;\mathrm{pt}\right)\right] \end{aligned}$$

where:

- True Positive Count: preoperative images suggested potential operability, and complete cytoreduction was achieved (i.e., beneficial surgeries);
- False Positive Count: preoperative images suggested potential operability, but surgical treatment was unsuccessful, or omitted for obvious reasons (e.g., inoperable disease or no evidence of disease by other modality);
- N: total number of patients;
- pt: probability threshold at which one would choose to treat.

Since conventional imaging suggesting possible operability was an initial inclusion criterion, it functioned as the “treat all” strategy in our main DCA. To address concerns that a hypothetical “treat all” comparator may overestimate the incremental value of PET/CT in this nested design, we performed a sensitivity analysis using an optimistic (“best-case”) conventional imaging strategy. In this analysis, conventional imaging–based selection was modelled under assumptions maximally favorable to conventional imaging:

1. all patients who ultimately achieved complete gross resection (R0) were assumed to have been correctly selected as surgical candidates (maximizing true positives), and
2. false-positive surgical selection attributable to conventional imaging findings was restricted to cases in which PET/CT revealed additional findings indicating inoperability that were not suspected on conventional imaging. Cases in which PET/CT findings were confirmatory of conventional imaging-suspected disease, or in which PET/CT clarified equivocal conventional imaging findings as benign, were not counted as selection errors.

This “best-case scenario” was used as a bounding analysis to evaluate whether the direction of net benefit in the decision curve analysis was robust to highly favorable assumptions for conventional imaging.

Survival analyses compared patients who achieved complete macroscopic resection following secondary cytoreductive surgery (SDS_R0 group) with patients who did not undergo secondary cytoreductive surgery after recurrence, or incomplete resection has been achieved (OTHER group). Survival distributions were estimated using the Kaplan–Meier method and compared using the log-rank test. Median survival times with 95% confidence intervals were reported.

## Results

### Patient selection

**Fig. 1:**
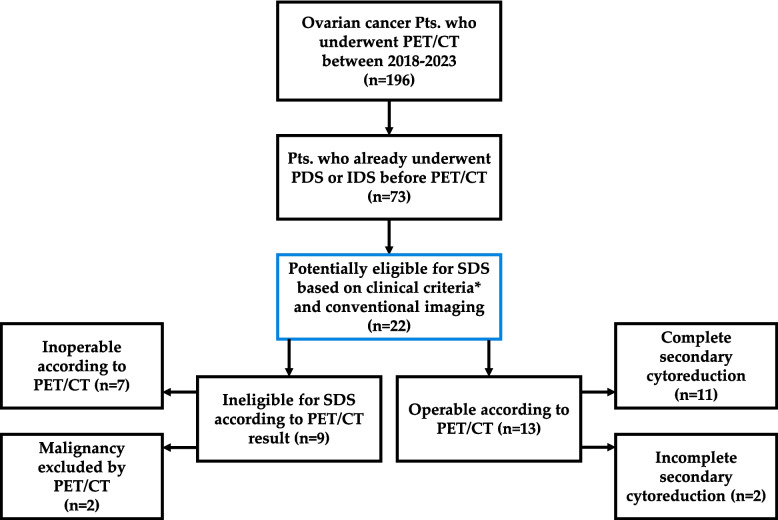
Flow diagram of study design and patient selection. The blue box represents the included patients in this study. * Clinical inclusion criteria are listed in the 2.1 Eligibility criteria section

A flow chart illustration of the study design is seen in Fig. 1.

A total of 196 patients with ovarian cancer underwent [^18^F]FDG PET/CT imaging at our institution between January 1 st, 2018, and December 31 st, 2023 for various indications (follow-up for contradictory radiological results, confirmation of therapy, elevation of CA 125 without radiological sign, preoperative assessment for primary and secondary debulking surgeries if necessary, etc.). Of these, 73 patients had already undergone a successful primary debulking surgery (PDS) or interval debulking surgery (IDS) before the PET/CT.

Twenty-two of these patients had a suspected recurrence, and were potentially eligible for secondary cytoreductive surgery (SCS) based on clinical assessment detailed in the 2.1 Eligibility criteria section and according to conventional imaging findings. These patients were included in our cohort.

In all 22 cases included in our final data analysis, the preoperative PET/CT examination was performed within 3 months of SCS. PET/CT imaging reclassified nine of these patients as ineligible for surgery: seven due to extensive or unresectable disease and two due to the absence of detectable malignancy. Importantly, the decision to forgo surgery in these cases was made by a multidisciplinary tumor board and was not based on PET/CT findings alone, but rather on an integrated assessment of clinical status, laboratory parameters, and imaging findings. Consequently, surgery was not withheld solely on the basis of PET/CT in situations where complete cytoreduction was considered potentially achievable. The remaining 13 patients were considered operable based on PET/CT findings and underwent SCS. Among these, complete cytoreduction was achieved in 11 cases, while 2 patients had incomplete cytoreduction.

### Patient characteristics and demographics

Patient characteristics and demographics are shown in detail in Table 1.

**Table 1:** Characteristics and demographic data of the enrolled patients.

Pt. #	Histology	Staging	Progression-free interval following prior surgery (months)
#1	HGSOC	III/A	18 months
#2	Undifferentiated carcinoma	III/C	38 months
#3	HGSOC	II/B	19 months
#4	HGSOC	IV/B	48 months
#5	HGOSC	III/C	19 months
#6	HGSOC	II/A	84 months
#7	HGSOC	III/C	35 months
#8	HGSOC	III/A	14 months
#9	HGSOC	III/C	31 months
#10	LGSOC	III/B	31 months
#11	HGSOC	III/C	26 months
#12	HGSOC	IV/B	17 months
#13	clear cell carcinoma	I/A	27 months
#14	adult-type granulosa cell tumor	IV	30 months
#15	HGSOC	III/B	25 months
#16	HGOSC	III/C	42 months
#17	HGSOC	I/C	48 months
#18	HGSOC	III/C	38 months
#19	HGSOC	I/A	186 months
#20	HGSOC	III/C	39 months
#21	endometrioid adenocarcinoma	I/C	N/A
#22	HGSOC	III/B	N/A

Progression-free interval after previous surgery was not applicable (N/A) for patients with no disease recurrence

The median age was 55.5 years (range, 35–82 years) at the time of the diagnosis and 58 years (range, 40–85 years) at the time of the SDS. The median progression-free interval following the previous debulking surgery was 31 months (range, 14–186 months). 16/22 (73%) patients were diagnosed at an advanced stage (FIGO III or IV), while 6/22 (27%) had an early-stage disease (FIGO I or II) at the time of diagnosis.

Histopathology confirmed HGSOC in 17/22 (77%) patients. Other histological subtypes that were present in the remaining five (23%) patients were undifferentiated carcinoma, low-grade serous ovarian cancer (LGSOC), clear cell, granulosa cell tumor, and endometrioid carcinoma.

### Performance of [^18^F]FDG PET/CT

Operability status based on the different modalities and surgical outcomes of the enrolled patients is shown in Table 2.

**Table 2:** Operability status according to preoperative conventional imaging and PET/CT findings, as well as surgical outcomes of the included patients.

Pt. #	Suggestion based on conventional imaging and clinical data	PET/CT suggestion	SCS result	Time between modalities
#1	potentially operable	potentially operable	complete	4 weeks
#2	potentially operable	potentially operable	complete	5 weeks
#3	potentially operable	potentially operable	complete	6 weeks
#4	potentially operable	potentially operable	complete	4 weeks
#5	potentially operable	potentially operable	complete	5 weeks
#6	potentially operable	potentially operable	complete	7 weeks
#7	potentially operable	potentially operable	complete	4 weeks
#8	potentially operable	potentially operable	complete	7 weeks
#9	potentially operable	potentially operable	complete	3 weeks
#10	potentially operable	potentially operable	complete	3 weeks
#11	potentially operable	potentially operable	complete	7 weeks
#12	potentially operable	potentially operable	incomplete	4 weeks
#13	potentially operable	potentially operable	incomplete	4 weeks
#14	potentially operable	definitively inoperable	no surgery	8 weeks
#15	potentially operable	definitively inoperable	no surgery	8 weeks
#16	potentially operable	definitively inoperable	no surgery	6 weeks
#17	potentially operable	definitively inoperable	no surgery	2 weeks
#18	potentially operable	definitively inoperable	no surgery	7 weeks
#19	potentially operable	definitively inoperable	no surgery	5 weeks
#20	potentially operable	definitively inoperable	no surgery	1 week
#21	potentially operable	negative for malignancy	no surgery	6 weeks
#22	potentially operable	negative for malignancy	no surgery	6 weeks

Time intervals between conventional imaging and PET/CT are also shown

All 22 patients enrolled in our study were considered potentially eligible for SDS, meaning that lesions with clear malignancy seen on these images were considered resectable. However, only 13 of these patients (Pt. #1–13) were considered operable based on PET/CT imaging (Table 2).

**Fig. 2:**
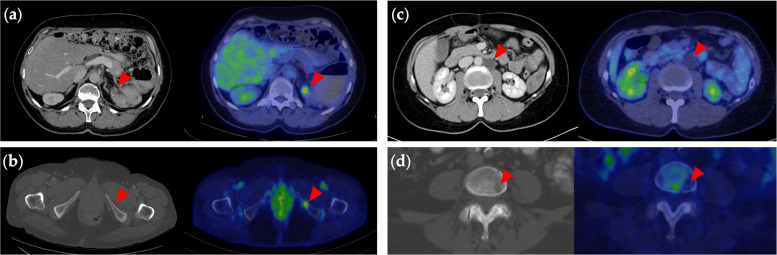
Selected cases demonstrating the added value of PET/CT over conventional imaging in the preoperative evaluation of recurrent ovarian cancer. PET/CT revealed an additional (**a**) lymph node metastasis (Pt. #7) and (**b**) a bone metastasis (Pt. #19) not identified on contrast-enhanced CT. Suspicious lesions seen on CT, such as (**c**) para-aortic cystic lesion (Pt. #21) and (**d**) lytic vertebral lesion (Pt. #8), were found to be non–FDG-avid on PET/CT, indicating benign etiology

Recurrent malignancy was ruled out by PET/CT in two patients (Pt. #21 and #22) (Fig. 2c). In these two patients with lesions suspicious for recurrence on conventional imaging, PET/CT did not demonstrate a metabolically active malignant disease. Pt. #21 and #22 were subsequently followed up to this date for 3 and 6 years respectively, during which no clinical, biochemical, or imaging evidence of disease recurrence was observed, confirming the non-malignant nature of the initially suspected findings. Seven patients (Pt. #14–20) were considered inoperable based on PET/CT findings (Fig. 2b). It means that PET/CT findings contradicted conventional imaging findings regarding resectability in 9 patients (Pt. #14–22). The reasons for inoperability were peritoneal, hepatic, bone, muscle, or unresectable lymph node involvement detected by PET/CT, and confirmed by follow-up (Table 3). In two patients (Pt. #14 and #16), PET/CT demonstrated supradiaphragmatic lymph node involvement. Following multidisciplinary tumor board discussion, including thoracic surgical consultation, these patients were considered ineligible for SDS due to the location of the lymph nodes. The patients who had unresectable metastases or no evidence of disease identified did not undergo SDS. PET/CT examination was, therefore, able to prevent unbeneficial surgical treatment and unnecessary morbidity in these patients.

**Table 3:** Additional findings or clarifications provided by PET/CT compared to conventional imaging.

Pt. #	Additional finding/clarification on PET/CT
#1	Not found
#2	Not found
#3	Not found
#4	Not found
#5	Not found
#6	Not found
#7	(+) Epigastric lymph node metastasis (new finding)
#8	(−) Lytic vertebral lesions (follow-up confirmed)
#9	Not found
#10	Not found
#11	Not found
#12	Not found
#13	Not found
#14	(+) Supradiaphragmatic inoperable lymph node involvement (new finding)
#15	(+) Muscular metastases (confirmed)
#16	(+) Pancreatic, splenic and abdominal lymph node involvement (confirmed); (+) supradiaphragmatic inoperable lymph node involvement (new finding)
#17	(+) Hepatic metastasis (confirmed)
#18	(+) Recurrent pelvic lesions (confirmed); (+) peritoneal metastasis on liver surface (new finding)
#19	(+) Pelvic recurrence (confirmed); (+) metastatic lesions on the dorsal surface of the liver (new finding); (+) bone metastasis (new finding)
#20	(+) Peritoneal carcinosis (new finding)
#21	(−) Para-aortic cystic lesion (follow-up confirmed)
#22	(−) Lymph node involvement (follow-up confirmed)

Findings marked with ”(+)” indicate malignant lesions on PET/CT: either not seen on conventional imaging (new finding) or seen but verified as malignant by PET/CT (confirmed). Findings marked with “(−)” were suspicious for malignancy on conventional imaging but were clarified as likely benign based on the radiopharmaceutical accumulation pattern, later clinically confirmed by follow-up (follow-up confirmed)

SDS was performed in the remaining patients with favorable PET/CT results (Pt. #1–13), of whom complete cytoreduction was achieved in 11/13 patients (85%) (Pt. #1–11). This means that the PPV of PET/CT for predicting operability was 85% (95%CI: 55–98%).

Patients who eventually had SDS were considered potentially operable by both conventional imaging and PET/CT; however, PET/CT was able to provide a more accurate clinical picture of two patients (Pt. #7 and #8). In one of these patients (Pt. #7), an additional metabolically active lesion was found on PET images, which would have remained undetected preoperatively if only anatomical imaging had been performed (Fig. 2a). In the other patient (Pt. #8), lytic bone lesions seen on CT scans were confirmed to be benign by PET/CT, further supporting the patient’s eligibility for SDS (Fig. 2d). The benign nature of these lesions was later confirmed on follow-up as well (Table 3).

The median interval between conventional imaging and PET/CT was 5 weeks (range, 1–8 weeks) (Table 3).

**Fig. 3:**
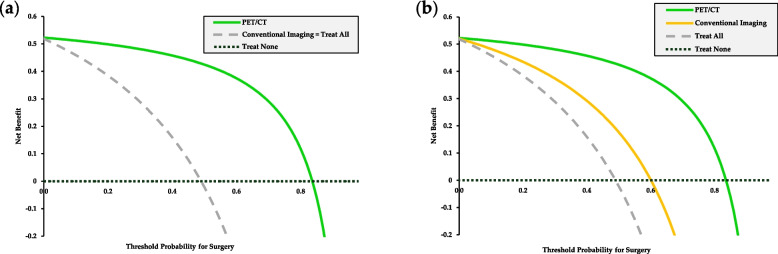
Decision curve analysis of PET/CT-based patient selection vs. conventional imaging-based patient selection for SCS. **a** Since operability could not be ruled out for certain with conventional modalities in any of the enrolled patients, it was equivalent to the “treat all” strategy. **b** An optimistic, best-case conventional imaging strategy is included as a bounding sensitivity analysis alongside treat-all and treat-none reference strategies

Decision curve analysis (DCA) was conducted to evaluate the clinical utility of PET/CT in selecting patients for SCS. Based on the previously presented data, both PET/CT and conventional imaging identified 11 true positives—patients who underwent surgery and achieved complete cytoreduction (Pt. #1–11). However, the number of false positives differed between the two modalities: PET/CT yielded 2 false positives (patients selected for surgery who did not achieve complete cytoreduction; Pt. #12 and #13). At the same time, conventional imaging would have resulted in 11 false positive cases at most (Pt. #12–22). A superior net benefit was observed across a wide range of surgical threshold probabilities when PET/CT was used for surgical decision-making compared with conventional imaging-based patient selection and a “treat none” strategy as well (Fig. 3a). Under the best-case conventional imaging comparator, conventional imaging corresponded to 11 true positives (Pt. #1–11) and 7 false positives (Pt. #12, #13, #14, #16, #18, #19 and #20), and PET/CT retained a directional net benefit across clinically relevant decision thresholds (Fig. 3b).

### Hospitalization and survival data

The length of hospitalization after SCS, as well as PFS and OS of all included patients are presented in Table 4.

**Table 4:** Individual patient data showing length of hospital stay following secondary debulking surgery (SDS), progression-free survival (PFS), and overall survival (OS).

Pt. #	Length of stay following SDS (days)	PFS (months)	OS (months)
#1	6	10.8	63.1
#2	9	8.9	117.7
#3	9	83.3	102.2
#4	9	6.2	116.6
#5	8	29.7	160.2
#6	8	75.6	164.5
#7	9	5.2	83.2
#8	10	16.4	44.7
#9	6	74.7	111.1
#10	9	23.2	96.4
#11	10	40.3	76.1
#12	7	15.1	41.8
#13	9	8.7	42.9
#14	N/A	12.8	53.7
#15	N/A	14.6	67.9
#16	N/A	2.0	44.4
#17	N/A	0.8	92.9
#18	N/A	39.9	122.3
#19	N/A	11.6	204.2
#20	N/A	5.6	173.0
#21	N/A	no recurrence	85.5
#22	N/A	no recurrence	88.5

Length of stay was not applicable (N/A) for patients who did not undergo surgery. PFS and OS are reported in months. “No recurrence” indicates absence of disease progression

Of the 13 operated patients no major (i.e., Clavien-Dindo grade III or IV) complication occurred during or after the operation, and the median hospitalization time was 9 days (range, 6–10 days).

When comparing patients who achieved complete macroscopic resection (SDS_R0) with all other patients with recurrent disease (OTHER group), a significant difference was observed in progression-free survival (PFS), whereas overall survival (OS) did not reach statistical significance.

The median PFS in the SDS_R0 group was 23.2 months (95% CI: 2.8–43.6), compared to 11.6 months (95% CI: 3.1–20.1) in the OTHER group. The interquartile range (IQR) for PFS was 8.9–NA months in the SDS_R0 group (25th–75th percentiles), compared to 5.6–14.6 months in the OTHER group. The difference in PFS was statistically significant (log-rank p = 0.033).

**Fig. 4:**
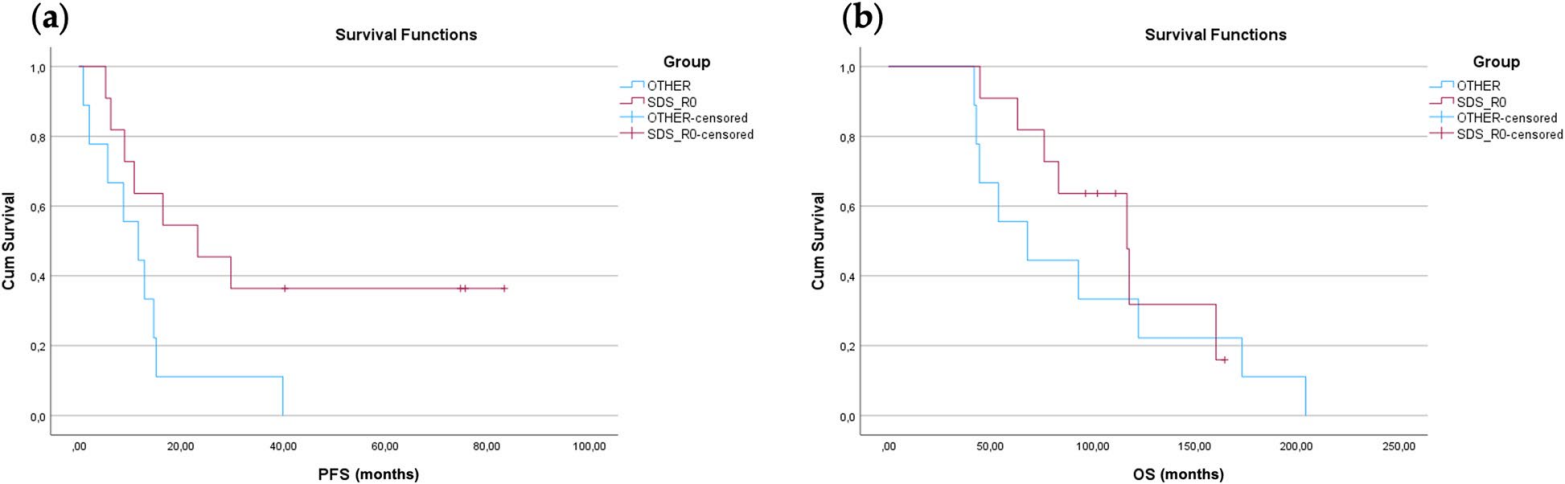
Kaplan–Meier curves comparing survival outcomes (**a** PFS,** b** OS) between patients achieving complete macroscopic resection following secondary cytoreductive surgery (SDS_R0) and patients who did not undergo secondary cytoreductive surgery because disease was deemed inoperable or incomplete resection has been achieved (OTHER group)

In contrast, although median OS was numerically longer in the SDS_R0 group (116.6 months; 95% CI: 79.3–153.9) compared with the OTHER group (67.9 months; 95% CI: 26.4–109.4), this difference did not reach statistical significance (log-rank p = 0.478). The OS interquartile range was 76.1–160.2 months in the SDS_R0 group versus 44.4–122.3 months in the OTHER group (Fig. 4).

These findings suggest that achieving complete cytoreduction is associated with a significant improvement in PFS, while the observed OS advantage did not reach statistical significance in the present cohort, likely due to limited sample size and wide confidence intervals.

## Discussion

Secondary cytoreductive surgery has a role in the treatment of recurrent ovarian cancer in specific clinical settings in case complete tumor reduction can be reached. Patient selection for this operation was investigated in previous clinical trials [6–9].

In the DESKTOP III trial, patient selection was based on the AGO score (ECOG performance status 0, ascites < 500 mL, and complete primary resection) [6]. Complete gross resection was achieved in 75% of cases, resulting in a significant overall survival benefit compared with chemotherapy alone (median OS 53.7 vs. 46.0 months; HR 0.75, p = 0.02) [6]. In contrast, the GOG-0213 trial used investigator judgment without an objective selection tool and found no survival advantage for surgery (median OS 50.6 vs. 64.7 months; HR 1.29, p = 0.08), possibly due to the lack of standardized selection criteria and the extensive use of bevacizumab in both arms [9]. The SOC-1 study combined the iMODEL score with PET/CT imaging to identify patients likely to achieve complete resection and demonstrated a significant progression-free survival benefit (median PFS 17.4 vs. 11.9 months; HR 0.58, p < 0.0001), with a trend toward improved overall survival [7].

[^18^F]FDG PET/CT is an imaging modality widely used in oncology for its superiority in detecting metastatic disease, with a higher sensitivity than most imaging modalities are capable of. Its advantages are seen not only in primary staging but also in the detection of tumor recurrence. Its use is becoming increasingly popular in gynecologic oncology as well [28–30]. Ovarian cancer is a type of malignancy that typically spreads via lymphatic dissemination and through direct implantation onto peritoneal surfaces. On contrast-enhanced CT, detectability of these metastases depends on lesion size and morphology as well as the attenuation contrast between the lesion and surrounding tissues, which is often insufficient for small lymph node involvement or peritoneal implants, making them difficult to distinguish from surrounding normal soft tissue on conventional imaging.

Despite its advantages, [^18^F]FDG PET/CT has well-recognized limitations that are particularly relevant when assessing operability in recurrent ovarian cancer. False-negative findings may occur in the presence of small-volume peritoneal implants or diffuse miliary disease, which can fall below the spatial resolution of PET. In addition, [^18^F]FDG avidity varies across histologic subtypes, with lower uptake commonly observed in low-grade serous, mucinous, and sex cord–stromal tumors, potentially reducing sensitivity in these settings [31]. Conversely, false-positive uptake may arise from inflammatory processes, postoperative changes, or benign reactive lymph nodes. These limitations underscore that PET/CT findings should be interpreted within the broader clinical and imaging context and not considered in isolation when determining surgical eligibility.

Even though whole body PET/CT has better sensitivity to detect certain metastases than conventional imaging modalities like contrast-enhanced CT [24, 32], its role in predicting surgical success in recurrent ovarian cancer patients has not been well established. There are only a handful of scientific articles proposing the potential use of PET/CT in the preoperative assessment of secondary debulking surgeries, and in many cases the other selection criteria besides PET/CT does not meet today’s standards since patient selection at the time of the study was not performed according to the latest guidelines [24–26, 33–37].

We recently conducted a systematic review and meta-analysis assessing the ability of PET/CT to predict complete cytoreduction in ovarian cancer. Even with limitations above, the results demonstrated that the positive predictive value (PPV) of PET/CT was consistently high for secondary cytoreduction, even higher and more consistent than in the primary cytoreductive setting—suggesting greater clinical utility of PET/CT in the context of SCS, although the number of studies for SDS is very limited [23].

In the past decade the surgical approaches towards both primary and secondary ovarian cancer surgery has evolved significantly, with increasing radicality for reaching the major goal of complete tumor reduction. This expresses the need to reassess the use of modern imaging modalities like PET/CT in today’s setting.

In this current study, we aimed to investigate the added value of PET/CT in the selection of patients for SDS. Patient enrollment was done following the current international guidelines. According to preoperative CT and MRI images, 22 patients were considered eligible for SDS, while only 13 patients were operable based on preoperative PET/CT.

Nine patients were ineligible according to PET/CT, the reasons being either a more advanced disease extent due mainly to peritoneal, hepatic, and bone involvement, or the exclusion of a potential recurrence. All decisions to omit surgical intervention were based on a multidisciplinary tumor board decision taking the PET/CT results into account as well, and SCS was not pursued when complete resection was considered unachievable. Notably, this included a patient with an adult-type granulosa cell tumor, a histologic subtype generally characterized by lower [^18^F]FDG avidity, in whom PET/CT nevertheless demonstrated intense tracer uptake and revealed disease extent incompatible with complete cytoreduction. This observation highlights that [^18^F]FDG PET/CT may be helpful in assessing disease extent in selected non-HGSOC subtypes in the real-world clinical practice, in accordance with guidelines.

Of the patients still considered operable, 11 underwent complete cytoreduction, and only 2 had an unsatisfactory surgical outcome. This means the PPV of PET/CT for predicting complete tumor resection was 85%, consistent with our previous meta-analysis.

Moreover, PET/CT showed a greater net benefit across a wide range of surgical probability thresholds compared with conventional imaging. This was true even when a “best-case scenario” was applied that represents an optimistic bound rather than a true reconstruction of clinical decision-making, and therefore likely underestimates the error rate of conventional imaging; nevertheless, it was included to avoid overstating the incremental value of PET/CT.

Our results therefore suggest that [^18^F]FDG PET/CT has a reasonable potential to become a reliable modality for assessing operability and identifying patients who are very likely to have a beneficial surgical outcome in the near future. However, prospective, multi-centric studies are required to overcome the limitations of this current study.

### Limitations

The main focus of this study was to evaluate the added benefit of PET/CT in surgical decision-making, and thus only patients undergoing preoperative PET/CT were enrolled. As a result, the overall value of conventional imaging modalities in selecting patients for SCS was not investigated and cannot be determined. A further limitation is that patients deemed inoperable by PET/CT did not undergo SCS, and it was presumed that they would have undergone incomplete cytoreduction. Because of this, true negative (i.e., considered unresectable and incomplete cytoreduction would have been achieved) and false negative (i.e., considered unresectable but complete cytoreduction could have been achieved) cases could not be differentiated, which could result in verification bias. Thus, no negative predictive value (NPV) could be calculated. All CT/MRI examinations were performed within two months before PET/CT. Although unlikely, interval disease progression cannot be excluded and may have influenced apparent operability, introducing a potential timing-related bias. These limitations are due to the retrospective nature of this study, and therefore, we emphasize the need for further prospective validation in multicenter trials on this topic. The median progression-free interval following the previous debulking surgery was 31 months (range, 14–186 months), which is longer than usual. We believe this can be explained by the strict inclusion criteria of our research, which allowed only patients who had undergone a prior complete resection and had solitary or oligometastatic lesions to be eligible.

## Conclusions

Our single-center data reinforce findings from our previously published meta-analysis and demonstrate a high PPV (85%) of PET/CT in patient selection for SCS, meaning that surgical treatment is likely to be beneficial for those, whose preoperative PET/CT result indicates complete resectability. PET/CT examination—as part of surgical planning—may also be useful in identifying inoperable lesions not visible on other modalities or to exclude malignancy in cases of an inconclusive conventional imaging report. Further prospective clinical studies are needed in this topic.

## Supplementary Information

Supplementary Material 1.

## Data Availability

All data generated or analysed during this study are included in this published article and its supplementary information files.

## Abbreviations

PET: Positron emission tomography
CT: Computed tomography
MRI: Magnetic resonance imaging
[^18^F]FDG: 2-Deoxy-2-[^18^F]fluoro-D-glucose
SCS: Secondary cytoreductive surgery
SDS: Secondary debulking surgery
FIGO: Federation of Gynecology and Obstetrics staging system
ECOG: Eastern Cooperative Oncology Group performance status
BMI: Body mass index
DCA: Decision curve analysis
Pt.: Patient
PDS: Primary debulking surgery
IDS: Interval debulking surgery
HGSOC: High-grade serous ovarian cancer
LGSOC: Low-grade serous ovarian cancer
PPV: Positive predictive value
NPV: Negative predictive value
